# Developmental Exposure to Low Concentrations of Methylmercury Causes Increase in Anxiety-Related Behaviour and Locomotor Impairments in Zebrafish

**DOI:** 10.3390/ijms222010961

**Published:** 2021-10-11

**Authors:** Lilah Glazer, Caroline H. Brennan

**Affiliations:** 1 Nanchang Joint Programme, School of Biological and Chemical Sciences, Queen Mary University of London, London E1 4NS, UK; 2 Department of Biological and Experimental Psychology, School of Biological and Chemical Sciences, Queen Mary University of London, London E1 4NS, UK; c.h.brennan@qmul.ac.uk

**Keywords:** zebrafish, methylmercury, developmental neurotoxicity, behaviour, anxiety, locomotor activity, HPI-axis, dopaminergic system

## Abstract

Methylmercury (MeHg) is a ubiquitous pollutant shown to cause developmental neurotoxicity, even at low levels. However, there is still a large gap in our understanding of the mechanisms linking early-life exposure to life-long behavioural impairments. Our aim was to characterise the short- and long-term effects of developmental exposure to low doses of MeHg on anxiety-related behaviours in zebrafish, and to test the involvement of neurological pathways related to stress-response. Zebrafish embryos were exposed to sub-acute doses of MeHg (0, 5, 10, 15, 30 nM) throughout embryo-development, and tested for anxiety-related behaviours and locomotor activity at larval (light/dark locomotor activity) and adult (novel tank and tap assays) life-stages. Exposure to all doses of MeHg caused increased anxiety-related responses; heightened response to the transition from light to dark in larvae, and a stronger dive response in adults. In addition, impairment in locomotor activity was observed in the higher doses in both larvae and adults. Finally, the expressions of several neural stress-response genes from the HPI-axis and dopaminergic system were found to be disrupted in both life-stages. Our results provide important insights into dose-dependent differences in exposure outcomes, the development of delayed effects over the life-time of exposed individuals and the potential mechanisms underlying these effects.

## 1. Introduction

Methylmercury (MeHg) is a neurotoxic contaminant generated through methylation of the heavy metal mercury in aquatic environments, where it then enters the aquatic food chain. Human exposure to MeHg occurs mainly in low doses through consumption of contaminated fish and seafood [1]. Human prenatal exposure to low levels of MeHg was linked to early-life neurotoxicity outcomes, including deficits in motor function [2,3], and lasting neurobehavioural impairments [4,5,6,7]. Behavioural impairments associated with MeHg exposure include reduced memory, language skills, and visuospatial perception [3,8], decreased IQ [9,10], anxiety [11], attention deficits and hyperactivity (ADHD) [12], and autism [13,14,15,16].

Most recent studies of MeHg neurotoxicity using experimental models have focused on adverse outcome pathways and behavioural effects of developmental low dose exposure. In rodents, pre- and perinatal exposures to low-doses of MeHg resulted in adverse emotional, cognitive and neuromotor effects in juveniles and adults, including depression-like behaviour [4,17,18], deficits in motor coordination and locomotor activity [19] as well as learning disability [20,21], and memory impairment [22]. In one study, the behavioural effects (learning deficits) emerged long after the leveling-off of brain MeHg levels [23]. In larval zebrafish, exposure of embryos to sub-lethal levels of MeHg resulted in reduced swimming activity [24,25] and impaired prey capture ability [24]. In adult zebrafish, developmental exposure was shown to cause learning and memory impairment [26], hyperactivity [27], and reduced responsiveness to startling visual cues with parallel retinal electrophysiological deficits [28].

Several cellular mechanisms have been found to be disrupted in the brain following developmental MeHg exposure, including the glutathione antioxidant system, intracellular calcium homeostasis, and the glutamatergic [29,30,31] and dopaminergic (DA) [32] neurotransmitter systems. Disruption of the endocrine hypothalamic-pituitary-adrenal (HPA) axis, involved in stress-response, has also been suggested as a mechanism involved in MeHg developmental neurotoxicity [33]. However, there is still a large gap in our ability to link adverse behavioural outcomes to specific neurodevelopmental perturbations, as well as in our understanding of the mechanisms underlying long-term behavioural effects of early exposure.

Many of the above-described behaviours and molecular mechanisms are linked to or interact with stress and anxiety-related behaviours. Yet, despite the large number of studies conducted to date on the various neurobehavioural effects of low-level developmental MeHg exposure and their potential underlying mechanisms, little is known about the relationship between such exposures and anxiety-related behaviours. In the current study, we tested the hypothesis that low-dose embryonic MeHg exposure disrupts the normal development of stress-response neurological pathways, causing long-term alteration of anxiety-related behaviour. For this purpose, zebrafish embryos were exposed to low doses of MeHg throughout embryonic development and the behavioural effects of the exposure were tested in larvae, shortly after exposure termination using the light/dark locomotor activity test, and in adult fish using the novel tank dive test and sensorimotor tap test. The behavioural testing was followed by gene expression analysis in exposed larvae and the brains of developmentally exposed and behaviourally tested adult fish. We compared the expression of genes related to stress response and locomotor activity, including the HPI-axis genes *Glucocorticoid Receptor alpha (grα)*, *Mineralocorticoid Receptor (mr)* and *Corticotrophin Releasing Factor (crf)*, and the DA pathway genes *Dopamine transporter (dat1)* and *Dopamine receptor 2b (drd2b)*.

## 2. Results

### 2.1. Larval Light/Dark Locomotor Activity Test

**Figure 1:**
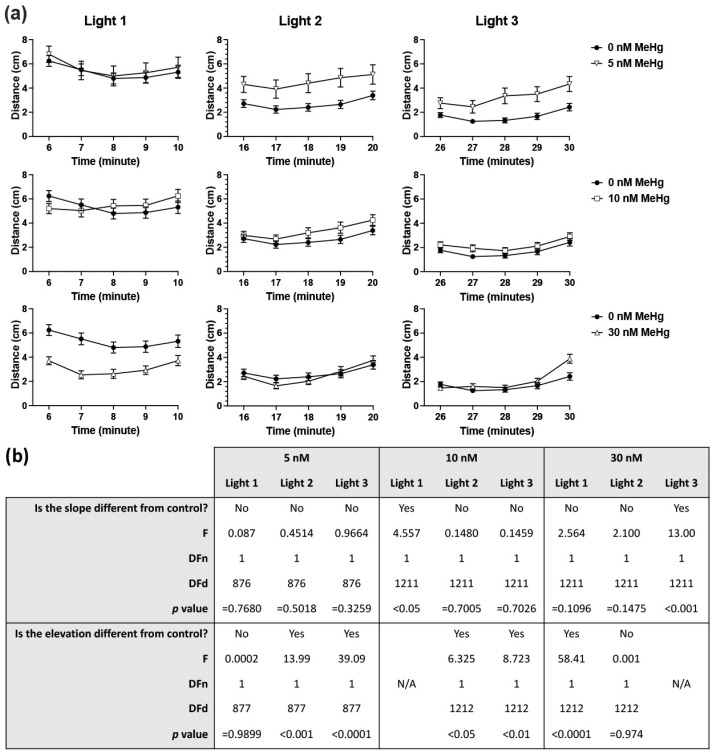
Six dpf larval locomotor activity in the three light phases of the light/dark locomotor activity assay and linear regression analysis. (**a**) Mean distance travelled per minute in each of the three light phases of the assay presented separately for each MeHg exposure dose compared to the control. (**b**) Linear regression analysis of difference in slope and elevation between each exposure dose and control. Subject numbers (N): 0 nM = 121; 5 nM = 55; 10 nM = 122; 30 nM = 122.

**Figure 2:**
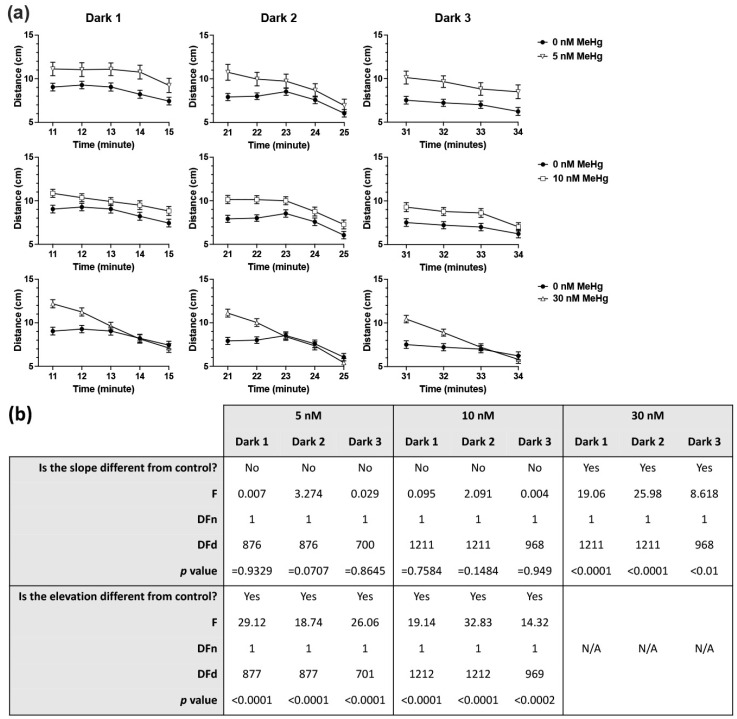
Six dpf larval locomotor activity in the three dark phases of the light/dark locomotor activity assay and linear regression analysis. (**a**) Mean distance travelled per minute in each of the three dark phases of the assay presented separately for each MeHg exposure dose compared to the control. (**b**) Linear regression analysis of difference in slope and elevation between each exposure dose and control. Subject numbers (N): 0 nM = 121; 5 nM = 55; 10 nM = 122; 30 nM = 122.

Developmental exposure of zebrafish embryos to MeHg resulted in altered behaviour at 6 dpf in response to alternating light and dark conditions (Figure 1, Figure 2 and Figure S1). Linear regression analysis of slopes and intercepts was conducted for each dark and light phase (excluding the initial dark acclimation period) comparing each MeHg exposure dose to the control group. During the light phases (Figure 1) the activity of each MeHg exposed group was affected differently. Exposure to 5 nM MeHg caused an increase in activity compared to control (i.e., change in linear elevation but not slope) during the second and third phases. The 10 nM exposed fish were similarly affected during the second and third light phases, however they also displayed an altered activity pattern during the first light phase, where the slope of activity was not significantly different from zero while the activity of the control group was higher during the first 2 min after the light turned on. Exposure to 30 nM MeHg caused significant reduction in activity during the first light phase and a change in activity pattern during the third light phase, with a steeper elevation in activity at the end of the phase. In all dark phases (Figure 2) it was found that exposure to the two lower doses of 5 and 10 nM MeHg caused increase in overall activity (elevation) of the larvae but no difference in the pattern of activity over time (slope). Conversely, exposure to the highest dose of 30 nM MeHg resulted in steeper activity slopes, with higher activity than control at the start of each dark phase followed by a fast decline in activity to control levels after the first 1–2 min.

### 2.2. Adult Behavioural Testing

When our embryonically treated fish reached adulthood, we conducted two behavioural assays; the novel tank dive test was used to identify alterations in anxiety-related response to mild stress in the form of a novel environment; and the tap test was used to detect altered sensorimotor response to an acute startle stimulus and is also used to assess habituation to repetition of the stimulus.

#### 2.2.1. Adult Novel Tank Dive Test

**Figure 3:**
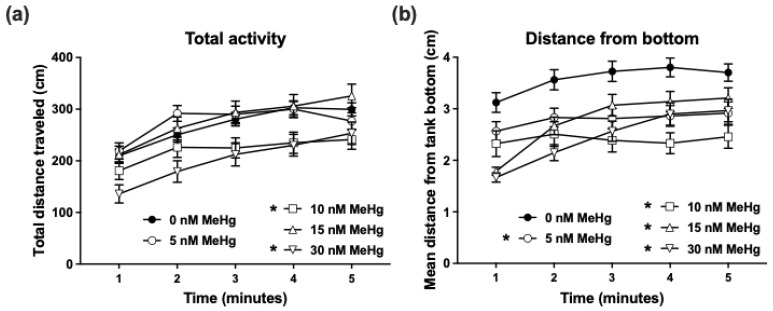
Novel tank dive test. Adult zebrafish that were developmentally exposed to 0 (control), 5, 10, 15, and 30 nM MeHg were individually placed in the testing tank (novel environment) and recorded for 5 min. Total activity (**a**) was calculated as the total distance travelled by the fish in each minute of the trial. Dive response (**b**) was calculated as the average distance of the fish from the bottom of the tank in each minute of the trial. Subject numbers (N): 0 nM = 111; 5 nM = 71; 10 nM = 52; 15 nM = 53, 30 nM = 57. Asterisks next to group names indicate significant difference from the control group.

In the novel tank dive test, the distance that the fish travel (total activity) in each minute of the 5-min trial is used as a measure of activity level (Figure 3A), while the distance of the fish from the bottom of the tank (distance-from-bottom) during each minute of the trial is used as a measure of anxiety-related dive response (Figure 3B). A repeated-measures two-way ANOVA of total activity throughout the trial revealed significant effects of minute (F_(3.173, 1076)_ = 57.01, *p* < 0.0001) and treatment (F_(4, 339)_ = 5.114, *p* < 0.001), such that the movement of the fish was increased from the beginning of the trial over time in all doses, with the 10 nM and 30 nM doses (but not 5 and 15 nM) causing a reduction in total activity of the fish compared to control. In addition, there was a significant interaction of treatment × time (F_(16, 1356)_ = 1.995, *p* < 0.05). Between-minute *t*-tests showed that, while the control (0 nM) group gradually increased their activity over the first 3 min and then their activity level plateaued, the activity of all MeHg treatment groups plateaued from minute 2 onwards. Similarly, distance-from-bottom was significantly affected by minute (F_(2.721, 922.3)_ = 32.30, *p* < 0.0001), with a gradual increase in distance over time, as well as by treatment (F_(4, 339)_ = 9.419, *p* < 0.0001), such that the distance-from-bottom in all MeHg treatments was decreased compared to the control. An interaction of treatment × time (F_(16, 1356)_ = 4.122, *p* < 0.0001) led to post hoc analyses showing that, while the distance-from-bottom in the control group plateaued from minute 2, in the 5 and 10 nM MeHg exposure groups there was no change in this parameter over time, and in the 15 and 30 nM MeHg exposure groups there was a gradual increase in distance-from-bottom in minutes 1 to 3 followed by plateau.

#### 2.2.2. Adult Startle Tap Test

**Figure 4:**
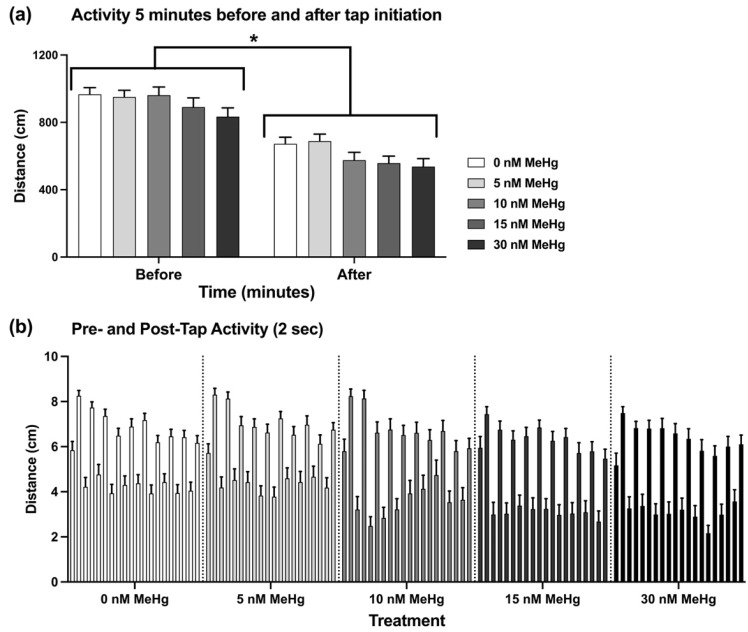
Tap test. Adult zebrafish were individually placed in cylindrical arenas, allowed a 10 min acclimation period followed by a sequence of 10 taps, one tap per minute. (**a**) activity in the 5 min before and after the initiation of tapping sequence. (**b**) activity of the fish in 2 sec pre- and post- each tap. The lower bars show the pre-tap activity and the higher bars show the post-tap activity. Subject numbers (N): 0 nM = 105; 5 nM = 72; 10 nM = 49; 15 nM = 51, 30 nM = 58. Asterisks indicate significant difference from the control group.

When comparing the overall activity of the fish in the 5 min after the tap sequence was initiated to the last 5 min of acclimation, we found a significant reduction in activity occurring in all treatment groups (F_(1, 330)_ = 291.4, *p* < 0.0001) (Figure 4A), with no significant effect of treatment. Tap response and habituation were analysed by comparing the distance that the fish travelled in the 2-sec before (pre-) and after (post-) each tap over the progression of the trial (across taps; Figure 4B). The pre-tap activity was used as a measure of the baseline activity of the fish throughout the trial, while the post-tap activity was a measure of the tap-response. Due to the large change in overall activity of the fish following the first tap, pre- and post-tap activity was only analysed in taps 2–10. Tap response was overall higher than the baseline activity throughout the trial, as was observed previously in similar protocols [34,35]. Pre-tap (baseline) activity was significantly affected by treatment (F_(4, 330)_ = 3.333, *p* < 0.05) such that activity was lower in all MeHg treatments except the lowest dose of 5 nM MeHg, compared to the control. There was no effect of tap number on activity. Post-tap activity was not affected by treatment, however there was a significant effect of tap (F_(7.455, 2460)_ = 10.39, *p* < 0.0001). Post hoc analysis found that the differences were mainly in reduction in activity from the second tap to the following taps in the control (0 nM), 5 nM and 10 nM MeHg groups, while in the two highest doses (15 nM and 30 nM MeHg) post-tap activity did not change throughout the trial. We also calculated the differences between the pre- and post-tap activities for taps 2–10 in each treatment, and compared them across treatments as a measure of the extent of tap response. Treatment was not found to have an effect on the calculated differences.

### 2.3. Larval and Adult Gene Expression

The results of the larval and adult behavioural testing indicated a potential two-component effect caused by the MeHg exposure; increase in anxiety-related response to stimuli such as alternating light and dark in larvae and introduction to a novel environment in adults; and reduced total activity as observed in the novel tank test and the tap test. Therefore, we decided to measure the expression of several genes involved in mediation of the stress-response and motor activity in zebrafish, including three HPI-axis genes and two dopamine-related genes.

#### 2.3.1. HPI-Axis Gene Expression

**Figure 5:**
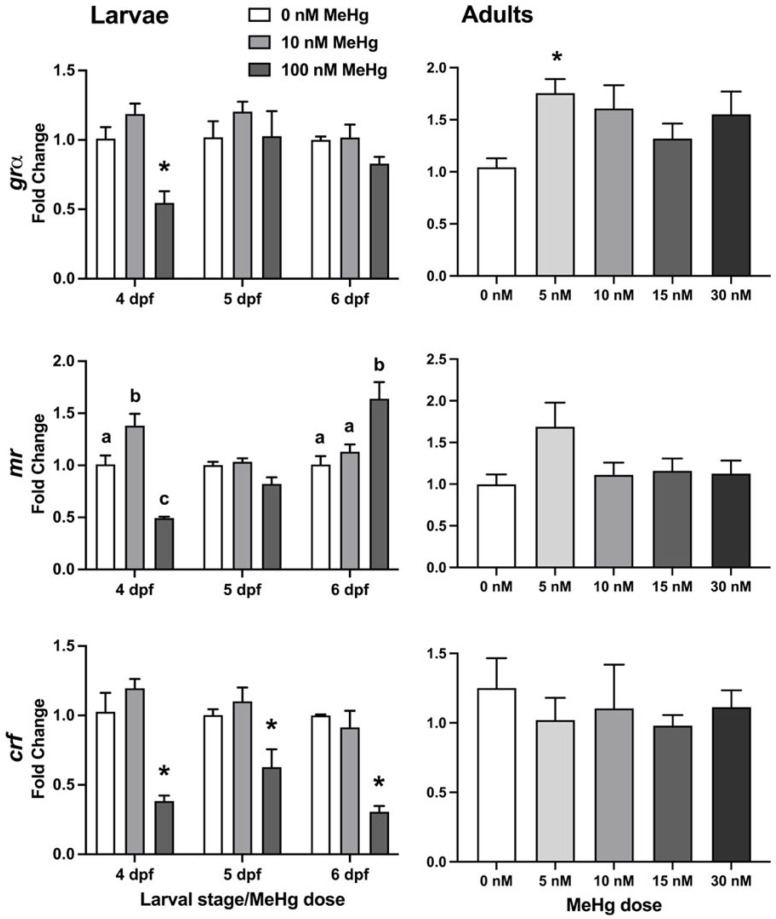
HPI-axis gene expression in larvae (left column) and adult brains (right column). Relative quantification of *grα* (top row), *mr* (middle row) and *crf* (bottom row) was measured in 4, 5, and 6 dpf larvae (left column, larval stage) and in the brains of developmentally exposed adult fish that were behaviourally tested, using RT-qPCR. *β-actin* was used as an internal standard. The ΔΔCt method was used to determine the fold change in gene expression. Subject numbers: larvae = 3; adult brains, 0 nM = 12–14, 5 nM = 6, 10 nM = 6, 15 nM = 7, 30 nM = 11–12. Significant differences from vehicle control are marked with an asterisk. Lower-case letters in the middle row indicate differences between means within 4 dpf and 6 dpf larvae. * *p* < 0.05.

Gene expression was analysed using real-time RT PCR for three HPI-axis genes (Figure 5): *grα, mr,* and *crf* in RNA extracted from whole larvae at 4 dpf (immediately after exposure termination), 5 and 6 dpf, and in the brains of behaviourally tested adult fish following MeHg developmental exposures. In larvae (Figure 5, left column), *grα* expression was found to be significantly affected by treatment (F_(2, 18)_ = 9.466, *p* < 0.005) but not by larval stage. Expression was lower in 100 nM exposed fish at 4 dpf compared to non-exposed fish, but not in any of the other stages or treatments. *mr* expression was significantly affected by both treatment (F_(2, 18)_ = 5.001, *p* < 0.05) and larval stage (F_(2, 18)_ = 13.16, *p* < 0.001), and there was an interaction between treatment and larval stage (F_(4, 18)_ = 20.64, *p* < 0.0001). Post hoc analyses found that *mr* expression was higher in 4 dpf larvae at the 10 nM dose compared to 0 nM, and lower at the 100 nM dose. The increase at 10 nM was not observed in 5 or 6 dpf fish. Interestingly, at 100 nM there was a gradual increase in *mr* expression, such that the low expression at 4 dpf was not observed at 5 dpf (there was no difference from the control) and at 6 dpf the expression level in the 100 nM exposed fish was higher than in the control. *crf* expression was significantly affected by treatment (F_(2, 18)_ = 47.77, *p* < 0.0001), such that it was lower in the 100 nM dose throughout the three larval stages, while there was no difference from control in the 10 nM exposed fish. In adult brains (Figure 5, right column), the expression of *grα* was affected by the exposure (*p* < 0.05), with a significant increase in expression in the 5 nM-exposed group, and a non-statistically significant increase trend in higher doses. There was no significant difference between any of the MeHg treatments and controls in *mr* or *crf* transcript expressions.

#### 2.3.2. Dopamine-Related Gene Expression

**Figure 6:**
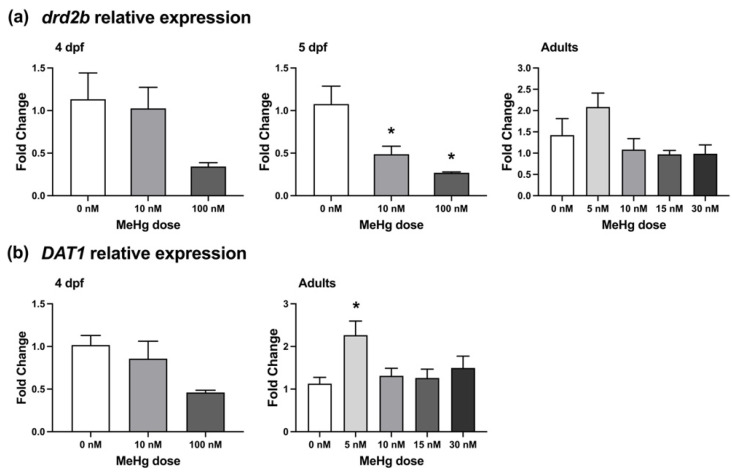
Dopamine-related gene expression in larvae and adult brains. Relative quantification of *drd2b* (**a**) and *dat1* (**b**) was measured in 4 dpf larvae and in the brains of developmentally exposed adult fish that were behaviourally tested, using RT-qPCR. *β-actin* was used as an internal standard. The ΔΔCt method was used to determine the fold change in gene expression. Subject numbers: larvae = 3; adult brains, 0 nM = 13, 5 nM = 6, 10 nM = 5–6, 15 nM = 7–8, 30 nM = 11–12. Significant differences from vehicle control are marked with an asterisk.

The expressions of two dopamine signalling-related genes, *drd2b* (Figure 6A) and *dat1* (Figure 6B), were measured in larvae and adult brains. The dopamine receptor *drd2b* was shown to be differentially expressed in response to MeHg exposure in zebrafish and mammalian models [32,36,37], while the expression of the dopamine transporter *dat1* was previously used to quantify the number DA neurons [37]. Levels of *drd2b* were not significantly altered by MeHg exposure when measured at 4 dpf, however by 5 dpf there was a significant reduction in expression of the transcript in both 10 nM and 100 nM treated larvae (*p* < 0.05). There was no significant effect of exposure on *drd2b* expression in the brains of adult fish that were behaviourally tested. Conversely, the expression of *dat1* was not significantly different in MeHg-exposed larvae compared to the non-exposed controls, however there was a significant increase in expression of the transcript in the brains of adult fish that were embryonically exposed to 5 nM MeHg (*p* < 0.05).

## 3. Discussion

Here, we used the zebrafish model to test the hypothesis that low-dose embryonic MeHg exposure causes long-term altered anxiety and impaired stress-response through disruption of normal neurological development, specifically of the HPI-axis and DA pathways. We found that exposures of zebrafish embryos to three sub-acute doses of 5, 10 or 30 nM MeHg caused significant and dose-dependent behavioural effects both in 6 days old larvae, shortly after the termination of exposure and several months later in adults, as well as significant immediate and delayed effects on both molecular pathways.

The dominant behavioural effect observed in larvae was an increased response to transitions from light to dark in all doses in the light/dark locomotor activity test, and overall higher activity in the two lower doses. The light/dark locomotor activity test is widely used to measure stress and anxiety in zebrafish larvae. Larvae commonly display hyperlocomotion following transition from light to dark phases, and rapid freezing followed by hypo-locomotion after transitions from light to dark phases [38,39]. Both locomotor responses are considered to be measures of stress response and increased anxiety. Conversely, other researchers use the light/dark assay as a general test to screen for early signs of neurotoxicity that can be further studied in more specialised assays or in later-life stages [34,35,40,41,42]. Similar protocols of zebrafish embryonic MeHg exposure and larval locomotor activity testing were conducted in only three other studies [24,33,43]. Samson et al. (2001) [24] exposed zebrafish embryos to a range of MeHg concentrations between 20–60 nM at different time windows from 0 to 72 hpf and measured larval spontaneous swimming activity. The authors found that exposure to 40 or 60 nM MeHg during most time windows resulted in reduced swimming activity by 8 dpf, while the 60 nM dose also caused delayed morphological defects and mortality by 9 dpf. Similarly, exposure of zebrafish larvae to 60 nM MeHg at 72–120 hpf by Huang et al. (2016) [43] resulted in reduction in spontaneous swimming activity at 6 and 7 dpf and a delayed appearance of morphological deformities. In the current study, we observed an increase in activity during dark phases in all MeHg doses. Interestingly, while in the lower doses the higher level of activity was maintained throughout the dark phases, in the highest dose of 30 nM this increase was only maintained in the first 1–2 min after which activity quickly decreased to control level. Furthermore, when conducting preliminary exposures to determine sub-acute dose-range for behavioural testing we found that exposure to 100 nM MeHg caused such marked hypo-locomotion that the larvae appeared to be almost unable to respond to the changes in illumination (data not shown). These results indicate a similar effect to the one found by Samson et al. (2001) [24] and Huang et al. (2016) [43] at MeHg doses higher than 30 nM. However, the main behavioural effect observed in our study at the lower doses of 5 and 10 nM MeHg was hyperlocomotion that was maintained throughout the dark phases of the assay compared to control, and to a lower extent also in the light phases. In the study conducted by Spulber et al. (2018) [33], zebrafish embryos were developmentally exposed to 2.5 nM MeHg from 2 hpf until 5 dpf and tested for spontaneous swimming activity and response to an acoustic startle at 5 dpf, and it was found that the exposure caused an increase in activity in both assays. Thus, in our experimental design, that included a wide range of sub-acute doses, we were able to provide important insight into the apparent discrepancy in observations across studies by teasing apart the two different behavioural effects caused by exposure to sub-acute doses of MeHg, i.e., an increase in locomotor activity caused by very low doses, and impairment in locomotor activity caused by higher doses. However, based on these results alone we cannot directly determine whether the MeHg exposure caused stress or anxiety at this early life-stage.

In adults, embryonic exposure to MeHg resulted in a stronger dive response observed in the novel tank dive test indicating heightened anxiety-related behaviour. The behavioural pattern of dive response and recovery in zebrafish is consistently observed in the novel tank dive test across research groups and zebrafish strains and is a result of the anxiety-promoting effect of introduction to a novel, potentially dangerous environment [44,45,46]. When introduced to an unfamiliar tank, zebrafish will usually dive towards the bottom of the tank and display reduced activity for a period of 1–2 min (may be longer in some strains). Dive recovery occurs over the following minutes, when the fish become more active and swim higher in the water column. The validity of the novel tank dive test in measuring stress response and anxiety-related behaviour in zebrafish was extensively studied and reviewed [47]. It was shown that the typical dive response is significantly reduced when the fish are tested in a non-novel tank [44]. Furthermore, the use of several different anxiolytic substances including nicotine [48], alcohol [49], and buspirone and diazepam [44] also significantly reduced dive response. In our study, MeHg exposure caused an increase in this anxiety-related behaviour at all doses. A recent study by Patel et al. (2019) [11] examined the relationship between very low-level mercury exposure during fetal development and a range of behaviour problems in children, and found significant positive association between gestational MeHg exposure and anxiety scores in the Behavioral Assessment System for Children (BASC-2) at 2–8 years. Our findings are further supported by the results of Xu et al. (2012) [27], where zebrafish embryos were exposed to 0, 10, 30, 100 or 300 nM MeHg at ~2–24 hpf. When the fish were 4 months old, an active avoidance training and testing protocol was conducted using a mild electric shock. While the control group was able to learn the task, all MeHg exposed groups were reportedly hyperactive, frequently swimming back and forth in the testing apparatus and unable to learn the task. The authors of the study concluded that MeHg exposure impaired the learning ability of the fish, however in light of our current results we suggest that the hyperactivity of the exposed fish may have been the direct result of the anxiogenic effect of the toxin and that the absence of learning was secondary to this effect. Thus, we show that zebrafish can reliably model at least some of the neurotoxic effects observed in humans, and provide important insight into the long-term timescale.

Stress response in zebrafish, as in other teleost fish, is mediated by the hypothalamus-pituitary-interrenal (HPI)-axis [50,51]. Activation of the HPI-axis involves release of corticotropin-releasing factor (CRF) by the hypothalamus, which in turn triggers release of adrenocorticotrophic hormone (ACTH) from the adenohypophysis. ACTH acts on the interrenal gland to release cortisol, which then binds to either glucocorticoid receptors (GRs) or mineralocorticoid receptors (MRs) throughout the body to activate transcription of stress-response genes. In zebrafish larvae, loss-of-function mutation in the GR (*gr^S357^*) eliminated the hyperlocomotion effect of MeHg in a spontaneous activity test [33]. In the same study, untreated *gr^S357^* mutants displayed a similarly higher activity during larval acoustic tap stimulation as did MeHg-treated wild-type larvae and their level of activity did not change as a result of MeHg exposure. We found that low-dose MeHg exposure did not affect GR gene-expression levels in larvae, however there was a significant increase in GR expression in the brains of adults treated with 5 nM MeHg and a trend toward increased levels in all other groups. In a recent study by Sireeni et al. (2020) [52], loss-of-function GR mutant larvae had altered locomotor activity in both the tap test and light/dark preference test, while adult fish displayed a strong increase in anxiety-related behaviour in an open field test. These behavioural effects are strikingly like those found in our study, leading us to support the hypothesis made by Spulber et al. (2018) [33] that MeHg exposure indeed affects GR signalling. However, further investigation is required in order to fully understand the nature of these effects, whether MeHg directly interacts with the GR protein, affects GR gene expression or alters GR signalling indirectly, and how the early life exposure to MeHg leads to a long-lasting change in GR expression and stress response in adults.

In addition to the anxiety-related effect, we found a negative effect on the total activity of adult fish exposed to 10 and 30 nM MeHg throughout the novel tank assay. Baseline locomotor activity was also reduced in MeHg doses higher than 5 nM in the tap test during pre-tap measurements. The dopaminergic (DA) system, and specifically dopamine receptors are known to be important in the control of locomotor activity [53], but more recently they have also been shown to be part of the stress response system, through regulation of the HPI-axis [54,55,56]. In a recent study in zebrafish, the expressions of the two dopamine D2 receptors, *drd2a* and *drd2b* were compared between groups of bold and shy adult fish that were classified based on their response to the novel tank dive test [55], and it was found that both receptors were expressed at higher levels in the bold fish indicating their importance for stress-coping mechanisms. Furthermore, sensitivity of the developing DA system to MeHg exposure was previously shown in a wide range of experimental models including zebrafish, rodents and in vitro cell cultures [32,36,37,43,57,58]. Therefore, we proceeded to test the hypothesis that MeHg-induced changes to dopamine signalling during development, specifically the expression of the dopamine receptor *drd2b,* contributed to the combination of behavioural effects (i.e., increased stress response and locomotor impairment) observed in our study in larvae and adults. In addition, we measured the expression of the dopamine transporter *dat1* as a quantitative marker for the abundance of DA neurons in our samples [37].

We found that the MeHg exposure caused a reduction in larval *drd2b* expression, without a change in the number of DA neurons, while in adults there was no change in the expression of *drd2b* and only a significant increase in *dat1* expression in the 5 nM-exposed group. A similar developmental effect of MeHg exposure on the DA system was observed in vitro in differentiating murine embryonic stem cells (mESCs) following exposure to MeHg for 6 days [37]. In these cells, the expression of *drd2*, along with *drd1a* and *drd3*, was downregulated. However, in this experiment the exposure also resulted in reduction in DAT protein activity measured in the cell culture, and decrease in amount of tyrosine hydroxylase (TH)-positive cell clusters, indicating a potential change in the neuronal composition within the in vitro culture. Our findings are complementary to two other studies in zebrafish, in which a relationship was found between developmental sub-acute MeHg exposure, larval locomotor activity and disruption of the DA system. In one study, a reduction in spontaneous swimming activity in larvae following MeHg-exposure was associated with reduced mitochondrial functionality in DA neurons in specific brain regions, but not with changes in DA neuron numbers [43]. In this study, the authors used transgenic zebrafish lines in which cells expressing the *dat* gene were fluorescently labelled. In a different study, dopamine levels were significantly decreased in MeHg-exposed embryos [58], at a dose which also resulted in impaired larval locomotor activity. Furthermore, alteration of dopamine-mediated locomotor activity was associated with reduction in dopamine D2 receptor binding in infant (PND 14) and juvenile (PND 21) rats exposed to low doses of MeHg throughout the perinatal period [32,36], and developmental exposure to MeHg was shown to cause decreased response to amphetamine, a dopamine-acting stimulant, in two-month old male mice [59].

Interestingly, studies using animal models so far mostly focused on the locomotor aspect of dopamine signalling and on relatively short-term effects in pre-adult life stages. Therefore, the importance of our findings is three-fold: (1) they support the hypothesis that developmental MeHg exposure disrupts dopamine signalling; (2) they provide a strong indication that MeHg-induced disruption of dopamine signalling is linked to a range of behavioural effects including locomotor activity and stress-response; (3) they suggest that the behavioural effects are life-long and continue well into adulthood. In our view, the DA system may be a key player in the adverse outcome pathway of MeHg neurotoxicity, and especially developmental neurotoxicity. This view was also expressed in a review article by Newland et al. (2015) [60] in the context of reduced intellectual performance in mammalian assays such as discrimination reversal and choice acquisition, where the authors hypothesised that even subtle gestational MeHg exposure can disrupt dopamine neurotransmission and cause long-lasting impacts on behaviour. Therefore, we call for additional investigation to be conducted in order to establish the nature of DA system disruption caused by MeHg during neurodevelopment and its long-term effects on multiple behavioural domains.

In conclusion, we found that developmental exposure to sub-acute doses of MeHg caused delayed increase in anxiety-related behaviour that was accompanied by long-term impairment in locomotor activity in the higher dose range. While impairments of both movement and fine motor functions have been extensively reported in MeHg exposed humans and animals, and are considered phenotypical hallmarks of MeHg toxicity and neurotoxicity [30,61], to date relatively little attention has been given to the relationship between MeHg exposure and impaired stress response and anxiety-related behaviour. Here, we suggest for the first time a potential link between the two phenotypical end-points. Our behavioural findings provide novel insights into dose-dependent differences in exposure outcomes and the development of delayed effects over the life-time of exposed individuals. These insights may prove invaluable especially in light of the high variation in exposure outcomes observed in different human populations at such low-levels doses [3,4,5,6,7,8,9,10,11,12,13,14,15,16]. Furthermore, we found short- and long-term changes in the expressions of HPI-axis and dopamine pathway-related genes, which strengthen the accumulating evidence for the involvement of both neural systems in shaping the behavioural consequences of early life MeHg exposure, although the potential mechanistic links require much further investigation. Our results provide a strong case for the advantages of using zebrafish as a reliable, valid and cost-effective model for robust investigations into life-long phenotypic and mechanistic outcomes of developmental MeHg exposure.

## 4. Materials and Methods

### 4.1. Fish Husbandry and Breeding

All the experiments were conducted using a local colony of Tuebingen (TU) wild-type strain of zebrafish (*Danio rerio*), maintained and bred in the Biological Services Unit (BSU) at Queen Mary University. All animal work was carried out following approval from the Queen Mary Research Ethics Committee, and under license from the Animals (Scientific Procedures) Act 1986. Care was taken to minimize the numbers of animals used in this experiment in accordance with the ARRIVE guidelines (http://www.nc3rs.org.uk/page.asp?id=1357, accessed on 4 October 2021). Where relevant, animals were sacrificed using terminal anaesthesia with tricaine (300 mg/L) followed by decapitation.

Adult zebrafish were held in mixed (females and males) groups at a density of ≤5 fish/l in 3 or 10 l tanks placed on a recirculating flowing water system (Tecniplast UK, London, UK), at a constant 14 h:10 h light:dark cycle. Water temperature was kept at 28 °C, and the fish were fed 3 times a day with flake food and brine shrimp. All fish were bred and reared in the aquarium facility at Queen Mary University of London, licensed by the UK Home Office. Fish water was prepared by dissolving sodium bicarbonate (0.9 mM), calcium sulphate (0.05 mM) and marine salts (0.018 g/L) (Sigma, Poole, UK).

Fertilized eggs, obtained by group breeding using in-tank inserts were collected, sorted and transferred to glass Petri dishes for rearing and chemical exposures. From 0 until 6 days post fertilization (dpf), embryos were kept in an incubator at a temperature range of 27–28 °C, and a 14:10 h light:dark cycle. At 6 dpf, larvae designated for adult testing were transferred to flow-through recirculating-system tanks, and the water level and diet were gradually adjusted to the age and size of the fish as described below.

Larvae were fed with ZMsystems ZM-000 high protein food particles (Tecniplast UK, London, UK) from 5 dpf–10 dpf, ZM-100 and paramecium from 11 dpf-14 dpf, and ZM-200 and brine shrimp from 14 dpf–30 dpf. At one month of age, animals were transferred into aquaria where they were fed ZMsystems flake food and brine shrimp.

### 4.2. Developmental Exposure to Methylmercury

Methylmercury(II) chloride (MeHg, CH_3_HgCl, CAS 115-09-3) standard solution in H_2_O was purchased from Alfa Aesar at a concentration of 1000 ppm (4 mM) and volume of 5 mL. A working dilution of 100 ppm (400 µM) was prepared in a glass vial and kept in 4 °C. Zebrafish embryos were exposed from approximately 5 h post fertilization (hpf) until 4 dpf to either fish water alone or concentrations of 5 nM–1 µM MeHg in fish water. Exposure timing was chosen to reflect as much as possible the equivalent time-frame of neurogenesis during mammalian prenatal period. In zebrafish, embryonic neurodevelopment is divided into primary and secondary neurogenesis. Primary neurogenesis takes place during early embryonic stages (pre-hatching) when the neuronal platform that enables larval locomotion and foraging develops [62]. Secondary neurogenesis starts approximately when hatching begins (~2 dpf). This stage of neural development is highly comparable to embryonic neurogenesis in amniotes, where major areas of the brain are formed and many neuronal systems, including learning and memory, basic emotions and precise motor control, begin to differentiate replacing primary pathways [62]. Therefore, we began exposure prior to formation of the neural crest (approx. 10 hpf) until late secondary neurogenesis, just before the larvae begin to feed (5 dpf) [63]. Several sets of exposure were conducted in which exposure doses were adjusted based on survival and behavioural outcomes, until a dose range was achieved where there was no effect on embryonic and larval survival, or on swimming capacity (i.e., sub-acute range). Each exposure set consisted of a water only treatment (negative vehicle control) and two or three doses of MeHg, and each treatment consisted of three dishes (replicates). Table 1 lists all exposure experiments conducted, larval survival and additional experimental details. Exposure to 1 µM caused 100% mortality within 24 h, and exposure to 300 nM caused 100% mortality by 4 dpf (data not shown). Larvae exposed to 100 nM showed marked overall reduction in activity at 6 dpf (data not shown). The final exposure doses used in larval behavioural analysis were 0, 5, 10, 30 nM MeHg, and in adult behavioural analysis were 0, 5, 10, 15, 30 nM MeHg.

Exposure protocol: at 5 hpf, embryos were sorted under a dissecting microscope, discarding unfertilized or abnormally developing embryos, then randomly and evenly distributed into glass Petri dishes (inner diameter 11 cm; depth 1.5 cm) at a density of 50 or 100 embryos per dish with 0.5 mL system water per embryo, and immediately exposed to the treatments detailed in Table 1. Exposure solution was replaced every 24 h until 4 dpf, when the embryos were rinsed twice with non-dosed fish water, transferred to clean plastic Petri dishes with non-dosed fish water, and placed in the incubator until larval testing at 6 dpf. All behaviourally tested larvae were euthanised immediately after testing.

### 4.3. Larval Light/Dark Locomotor Activity Test

Six days old larvae were subjected to a light/dark locomotor activity test. Larvae were sampled for testing out of the treatment dishes and euthanized immediately after testing. All exposure conditions were represented and balanced within each plate and across multiple plates. All behaviour testing was conducted between 9:00 and 17:00. All video recordings were analysed using the EthoVision XT^®^ tracking software (version 11.5, Noldus, Wageningen, The Netherlands). A track smoothing protocol was applied based on 10 samples before and after every sample point in order to exclude slight movements that might introduce noise to the calculations.

Testing procedure: larvae were placed into 48-well plates filled with 1 mL fish water in each well, one larvae per well, and tested for locomotor activity in response to alternating light and dark phases. Following a half hour acclimation on the bench in the testing room, the dishes were placed in a Zantiks MWP unit (Zantiks Ltd., Cambridge, UK) with a bottom screen (visible light source) and recorded for 35 min. The testing paradigm consisted of an initial 5 min acclimation period in the dark, followed by 3 cycles of 5 min light and 5 min dark. An infrared top-view camera recorded the plates during trials. Total distance moved is reported in cm per minute. Raw data for individual fish movement can be found in Supplementary Tables S1 and S2.

### 4.4. Adult Behavioural Test Battery

Developmentally exposed adult zebrafish were tested at the age of 4–10 months in two behavioural assays; novel tank dive test and the startle tap test, to evaluate emotional and motor functions. Each assay was conducted on a separate day. All testing was conducted between 9:30 and 17:00, and testing times were counterbalanced across all experimental groups. Each testing day began after the routine morning pellet feeding. Fish tanks designated for testing were removed from the flow-through system, transferred to the behaviour testing room, and let acclimate for 45–60 min. Fish water from the flow-through system was used in all testing apparatuses. Video recording in both assays was conducted using a DMK 23UX236 Imaging Source camera (The Imaging Source Europe GmbH, Bremen, Germany) with a H0514-MP2 lens (Computar, CBC Group LLC, Cary, NC, USA).

#### 4.4.1. Novel Tank Dive Test

Adult zebrafish were tested for novel environment response and recovery based on the method developed by Levin et al. (2007) [48] with modifications. The experimental set-up consisted of two adjacent 1.5-L plastic tanks (Pentair, MBK Installations Ltd., Nottinghamshire, UK) placed in front of an infra-red light-source and filled with 10 cm of fish water. Each tank was a trapezoid: 22.5 cm along the bottom, 27 cm at the top, 15.2 cm high and 16 cm along the diagonal side. Tank width was 6.9 cm at the top and tapered to 4.8 cm at the bottom. At the beginning of each trial two fish were individually placed in the testing tanks and recorded for 5 min. The tanks were video recorded from side-view and the recorded videos were analyzed using the EthoVision XT^®^ software (Noldus Ltd., Nottingham, UK).

Measurements extracted were total distance travelled in cm for each min of testing and the mean distance from the tank floor in cm per min. Raw data for individual fish movement can be found in Supplementary Tables S3 and S4.

#### 4.4.2. Adult Startle Tap Test

**Figure 7:**
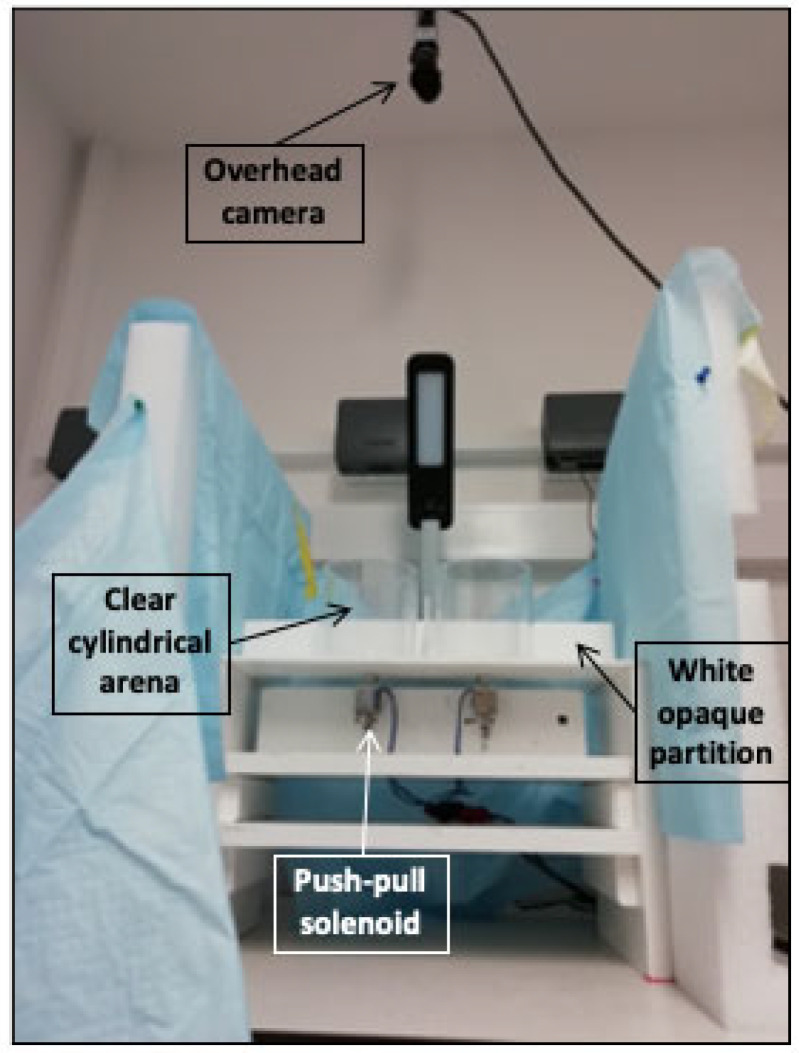
Side view of the adult tap-test experimental set-up. The apparatus was made of four clear cylindrical arenas attached to a flat white plastic surface with white opaque partitions separating the arenas. The plastic surface was placed on a wooden structure holding four push-pull solenoids centrally located under the four arenas. The apparatus was surrounded by Styrofoam sheets for visual isolation, and video recorded from above.

Sensorimotor startle response and habituation were tested using a custom-built apparatus and based on a protocol developed in the Levin Lab at Duke University [64,65], with modifications. The testing apparatus (Figure 7) consisted of a flat white 40 cm × 22 cm plastic surface. Attached to the flat surface were four clear cylindrical arenas, 9.2 cm in diameter, made of Plexiglas and arranged in a 2 × 2 setup. White opaque partitions separated the arenas, isolating subjects from each other to eliminate shoaling behaviour, and the whole apparatus was surrounded by Styrofoam sheets for visual isolation. Each arena contained 260 mL of fish water (40 mm depth) that were replaced after each trial. The plastic surface was placed on a wooden structure holding four 24-volt DC push-pull solenoids (JF-0826B; SourcingMap, San Bruno, CA, USA) centrally located under the four arenas, providing a sudden tap when activated. Timing of taps was programmed using the EthoVision XT^®^ software (Noldus Ltd.) that sent logic pulses at scheduled times to a second experiment–control computer via a parallel port connection. Software running in the control computer was used to activate the four-solenoid battery via solid-state relays. The apparatus was video recorded from above and the camera output was fed into the EthoVision XT^®^ software (Noldus Ltd.) for analysis.

The assay protocol was conducted over two consecutive days; acclimation day followed by testing day. On the acclimation day, the fish were individually placed in the arenas and recorded for 15 min without tap stimulus, then placed back in their home tank. On the testing day, the fish were individually placed in the testing arenas and the testing sequence was initiated. The testing sequence consisted of a 10 min acclimation period followed by 10 consecutive taps at 1 min intervals. Measurements extracted were total distance travelled in cm per the 2-s period before (pre) and after (post) each tap. The choice of pre- and post-tap 2-s time segments was based on pilot tests conducted in our lab during method development and found to provide consistent and sensitive measures of baseline and tap-response activity (data not shown). Raw data for individual fish movement can be found in Supplementary Table S5.

### 4.5. Whole Larvae and Adult Brain Sampling, RNA Extraction and Real-Time RT qPCR

Two types of samples were collected for RNA extraction, whole larvae shortly after embryonic exposure, and brains from adult fish that were embryonically exposed and behaviourally tested. Larval samples were collected at 4 dpf, 5 dpf and 6 dpf following exposure 5 (see Table 1). Pools of 10 larvae were sampled from each treatment replicate (3 replicates per treatment) into 1.5 mL tubes and snap frozen in liquid nitrogen. Brain tissue was dissected from adult fish after completion of behavioural testing, placed in 1.5 mL tubes and flash-frozen in liquid nitrogen. Samples were stored in −80 °C until total RNA isolation. Total RNA was extracted using TRIzol™ Reagent (Thermo Fisher Scientific Inc., Loughborough, UK) according to the manufacturer’s instructions. Complementary DNA was synthesized from total RNA using ProtoScript^®^ II First Strand cDNA Synthesis Kit (New England Biolabs, Hitchin, UK) from 1 mg total RNA. Quantitative real-time PCR was carried out using the *Power* SYBR™ Green PCR Master Mix (Applied Biosystems; Thermo Fisher Scientific Inc., Loughborough, UK) in a CFX Connect Real-Time PCR Detection System (Bio-Rad Laboratories Ltd., Watford, UK). The PCR conditions used were 95 °C for 5 min and 95 °C for 10 s/60 °C (primer annealing temperature) for 10 s/72 °C for 10 s (45 cycles). Table 2 lists the primers used to amplify the following transcripts: *β-actin*, *grα*, *mr, crf, dat1,* and *drd2b*. Each PCR reaction was run in duplicate and a no-template control reaction was run on each PCR plate. Melt curve analysis was performed at the end of each PCR run to ensure that no nonspecific products are amplified. Relative levels of transcript abundance were calculated using the 2^−ΔΔCt^ method (where ΔΔCt = [Ct_(GOI)_ − Ct_(β-actin)_]treated sample − [Ct_(GOI)_ − Ct_(β-actin)_]control average (GOI, gene of interest; Livak and Schmittgen, 2001) [66]. The individual fold-change for each replicate treatment sample was calculated against the non-exposed control average.

### 4.6. Statistical Analysis

All statistical analyses were performed in GraphPad Prism (GraphPad Software, Inc., San Diego, CA, USA; version 8.2.1). Prior to data analysis, outliers were identified using the Iterative Grubbs’ method and removed. Q-Q plots of residuals were used to visually assess data normality, and a log-log transformation was carried out where the data appeared to violate the assumption of normality. Significance was set at *p* < 0.05 for all linear regression and analysis of variance (ANOVA) tests and Dunnett’s post hoc comparisons. Raw behavioural data for individual fish movements can be found in the Supplementary Materials.

Analysis of larval locomotor activity at 6 dpf was carried out separately for each light and dark phase using a simple linear regression analysis to identify differences in slope or elevation between the control and each of the treated groups.

Adult tank dive test and tap test were analysed using repeated measures ANOVA with a Geisser-Greenhouse correction and Dunnett’s post hoc comparisons. Distance moved (cm per minute) or distance from the tank bottom (cm) were defined as the dependent factors. Treatment was defined as the between-subjects factor, and time as within-subjects factor and repeated measure in all behavioural tests.

Relative gene expression data were analysed separately for whole larvae and adult brains to determine significant differences between MeHg-exposed and control groups. Data was analysed with a one-way ANOVA, with treatment as the independent variable and relative fold change (2^−ΔΔCt^) as the dependent variable. Dunnett’s post hoc comparisons were performed to determine significant difference from the vehicle control.

## Figures and Tables

**Table 1:** List of MeHg exposure experiments.

Exposure	MeHg Concentrations	Number of Embryos Exposed	Survival at 6 dpf	Behaviour Testing	Comments
1-behaviour	0 nM	50 embryos × 3 dishes = 150 per treatment	>95%	Larval only	All euthanised at 6 dpf Due to technical difficulties- behaviour data was not included in the analysis
	30 nM		>95%		
	100 nM		>95%		
	300 nM		All dead by 6 dpf		
2-behaviour	0 nM	100 embryos × 3 dishes = 300 per treatment	1/3 dishes- 50% dead at 72 hpf 2/3 dishes- > 95%	Adult	5 nM- euthanised at juvenile stage due to experimental design
	5 nM		>95%		
	15 nM		>95%		
	30 nM		>95%		
3-behaviour	0 nM	100 embryos × 3 dishes = 300 per treatment	Low at 14 dpf in all groups	Larval only	Due to overall low survival at 14 dpf- all groups were euthanised at 3 weeks
	10 nM				
	30 nM				
4-behaviour	0 nM	100 embryos × 3 dishes = 300 per treatment	>95%	Larval and adult	30 nM- Due to low survival at juvenile stage, tanks 1 and 2 were pooled before adult testing
	5 nM		>95%		
	10 nM		>95%		
	30 nM		>95%		
5-Larval RNA	0 nM	50 embryos × 3 dishes = 150 per treatment	>95%	Non	5 larvae from each dish sampled for RNA extraction at 4, 5, 6 dpf Remaining larvae euthanised at end of experiment
	10 nM		>95%		
	100 nM		>95%		

**Table 2:** List of primers used in Real-Time RT qPCR.

Gene	Primer Sequences	Product Size (bp)
*Glucocorticoid Receptor alpha (grα)*	F 5’ ACTCCATGCACGACTTGGTG 3’ R 5’ GCATTTCGGGAAACTCCACG 3’	90
*Mineralocorticoid Receptor (mr)*	F 5’ CTTCCAGGTTTCCGCAGTCTAC 3’ R 5’ GGAGGAGAGACACATCCAGGAAT 3’	74
*Corticotrophin Releasing Factor (crf)*	F 5’ CGAGACATCCCAGTATCCAAAAAG 3’ R 5’ TCCAACAGACGCTGCGTTAA 3’	59
*Dopamine transporter (dat1)*	F 5’ GCCTGGTTTTACGGAGTGGA 3’ R 5’ GGAGGATTGAAGGTGGCGAA 3’	152
*Dopamine receptor 2b (drd2b)*	F 5’ GCACGGCCAGCATTCTTAATC 3’ R 5’ GAAAGCACCCAAACAACGGA 3’	136
*ß-actin*	F 5’ CGAGCTGTCTTCCCATCCA 3’ R 5’ TCACCAACGTAGCTGTCTTTCTG 3’	86

## Data Availability

The raw data from all behavioural assays are available in Supplemtary Materials.

## Supplementary Materials

The following are available online at https://www.mdpi.com/article/10.3390/ijms222010961/s1.
