# Supplementary material for: Developmental Exposure to Low Concentrations of Methylmercury Causes Increase in Anxiety-Related Behaviour and Locomotor Impairments in Zebrafish
Source: Int J Mol Sci. 2021 Oct 11;22(20):10961. doi: 10.3390/ijms222010961 (PMC8535691; doi:10.3390/ijms222010961)
Supplement: Supplementary file 1 [file ijms-22-10961-s001.zip › Figure S1_6 dpf larvae.pdf]

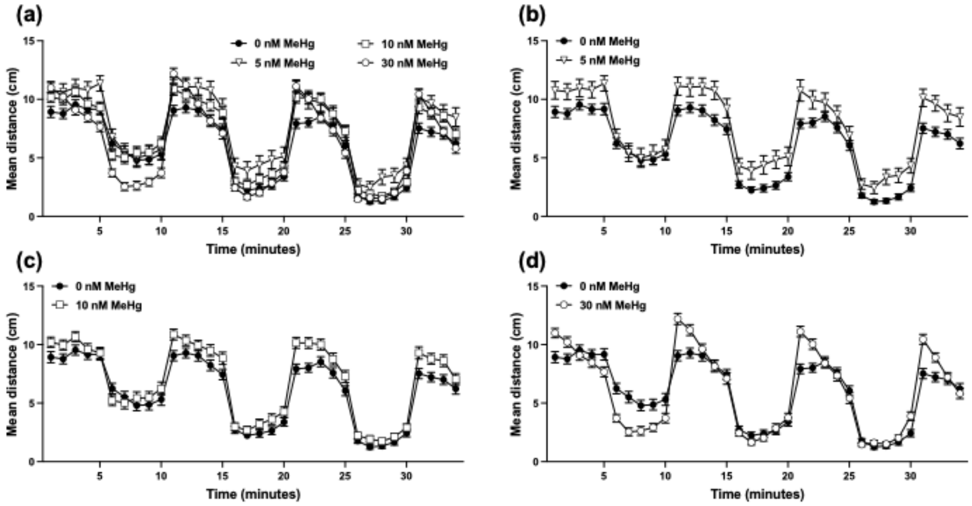

Figure S1. Six dpf larval locomotor activity in response to alternating light/dark conditions. At 6 dpf larval activity was recorded for 35 min, starting with 5 min in the dark (habituation), followed by three cycles of 5 min in the light and 5 min in the dark. Graphs present mean distance travelled per minute. (a) all experimental groups, (b) only 0 and 5 nM, (c) only 0 and 10 nM, (d) only 0 and 30 nM. Subject numbers (N): 0 nM = 121; 5 nM = 55; 10 nM = 122; 30 nM = 122.
